# Role of the Central Cholinergic System in the Therapeutics of Schizophrenia

**DOI:** 10.2174/157015908785777247

**Published:** 2008-09

**Authors:** Alvin V Terry, Jr

**Affiliations:** Department of Pharmacology and Toxicology, Medical College of Georgia, Augusta, Georgia, 30912 (AVT), USA

**Keywords:** Acetylcholine, muscarinic, nicotinic, receptor, cognition, psychosis.

## Abstract

The therapeutic agents currently used to treat schizophrenia effectively improve psychotic symptoms; however, they are limited by adverse effects and poor efficacy when negative symptoms of the illness and cognitive dysfunction are considered. While optimal pharmacotherapy would directly target the neuropathology of schizophrenia neither the underlying neurobiological substrates of the behavioral symptoms nor the cognitive deficits have been clearly established. Abnormalities in the neurotransmitters dopamine, serotonin, glutamate, and GABA are commonly implicated in schizophrenia; however, it is not uncommon for alterations in the brain cholinergic system (e.g., choline acetyltransferase, nicotinic and muscarinic acetylcholine receptors) to also be reported. Further, there is now considerable evidence in the animal literature to suggest that both first and second generation antipsychotics (when administered chronically) can alter the levels of several cholinergic markers in the brain as well as impair memory-related task performance. Given the well-established importance of central cholinergic neurons to information processing and cognition, it is important that cholinergic function in schizophrenia be further elucidated and that the mechanisms of the chronic effects of antipsychotic drugs on this important neurotransmitter system be identified. A better understanding of these mechanisms would be expected to facilitate optimal treatment strategies for schizophrenia as well as the identification of novel therapeutic targets. In this review, the following topics are discussed: 1) the central cholinergic system in schizophrenia 2) effects of antipsychotic drugs on central cholinergic neurons 3) important neurotrophins in schizophrenia, especially those that support central cholinergic neurons; 4) novel strategies to optimize the therapeutics of schizophrenia *via* the use of cholinergic compounds as primary (i.e., antipsychotic) treatments as well as adjunctive, pro-cognitive agents.

## INTRODUCTION

Schizophrenia is a chronic neuropsychiatric illness that affects approximately 1% of the world’s population. Core features of the disease [reviewed,^ 3^] include positive symptoms (e.g., hallucinations, delusions), negative symptoms (e.g., anhedonia, alogia, depression) and cognitive dysfunction (e.g., impaired working memory, attention, cognitive flexibility). This debilitating array of clinical symptoms commonly requires life-long therapeutic intervention. The primary therapeutic agents used for schizophrenia (i.e., the antipsychotic drugs) have been shown in multiple clinical trials to improve the behavioral (i.e., primarily positive) symptoms in most schizophrenic patients. However, the older, conventional agents (also referred to as typical or first generation antipsychotics) are limited by a number of adverse reactions including motor effects (e.g., Parkinsonian symptoms and tardive dyskinesia). Likewise, the newer agents (referred to as atypical or second generation antipsychotics) are now associated with several long term side effects including abnormal weight gain, development of diabetes mellitus, and hyperlipidemias [reviewed,^ 35^].

An important limitation to strategies for novel drug development is the relatively poor understanding of the neuropathology of schizophrenia. In postmortem brains of schizophrenic patients, evidence of reduced neuronal size, neuropil loss (particularly synaptic elements), decreased cortical,volume, and abnormal synaptic organization is substantial [39]. In addition, abnormalities in the neurotransmitters dopamine, serotonin, glutamate, and GABA (and their receptors) are also commonly implicated the neuropathology of schizophrenia. While the abnormalities described above are widely postulated to be neurodevelopmental in origin, the brains of antipsychotic-naïve schizophrenic patients have only rarely been analyzed. Thus, the possible contribution of chronic drug exposure to such neuropathological findings and neurotransmitter alterations is largely unexplored. In addition to the neurotransmitter receptor abnormalities described above, both the therapeutic actions and adverse effects of antipsychotic drugs have for decades been attributed to their relative affinities for specific neurotransmitter receptors [98]. The mechanism of action of conventional antipsychotics is most often attributed to their high affinity as antagonists at the dopamine (D_2_) receptor subtype in the CNS, whereas the mechanism of newer, second generation compounds is commonly attributed to a weaker affinity at D_2_ receptors combined with antagonist activity at other sites, such as the 5HT_2A_ serotonin receptor [reviewed,^ 66^]. It should be noted, however, that such receptor interactions occur almost instantaneously whereas many of the therapeutic effects and adverse reactions of antipsychotics can be observed only after chronic treatment periods. Thus, collectively, the information described above indicates that neither the neuropathology of schizophrenia nor the mechanism of action of effective therapeutic modalities is well understood. There is, therefore, a critical need for additional (i.e., more extensive) research to improve our understanding of both of these important subjects since the absence of such information limits decisions concerning optimal therapeutic strategies as well as the development of new treatments for schizophrenia. 

## THE CHOLINERGIC SYSTEM IN SCHIZOPHRENIA

Notwithstanding the neurotransmitter anomalies described above, it is also not widely appreciated that alterations in markers for the neurotransmitter, acetylcholine have been commonly reported in schizophrenia [80, 91]. Such data may be significant to both the psychotic symptoms and the cognitive deficits of the illness. Cholinergic deficits in basal forebrain structures and their projections in schizophrenia (see below) may be of particular importance to the cognitive dysfunction given their known functional roles in conscious awareness, and components of information processing, including attention, working memory, encoding, memory consolidation, and retrieval [36, 74]. 

It has been known for many years that anticholinergic compounds when administered in high doses can induce psychosis. For example, muscarinic antagonists such as scopolamine or atropine can evoke a psychotic state termed “anticholinergic syndrome” or “antimuscarinic psychosis” which shares many features with endogenous schizophrenia [18, 42, 56, 61, 64, 72, 73, 102, 105]. Other non-selective mAChR antagonists (e.g., Ditran) were also found to induce psychosis in normal humans and to exacerbate the symptoms of schizophrenia [68]. Further, the antimuscarinic agent, procyclidine, was reported to block the effects of the conventional antipsychotic flupenthixol on positive symptoms in schizophrenia patients [45]. 

For more than 30 years alterations in the acetylcholine synthesizing enzyme, choline acetyltransferase (ChAT), in schizophrenia have been reported [see ref 55]. Examples include ChAT alterations in the septum [60], nucleus accumbens, and pontine tegmentum [8, 46]. Elevated levels of choline, the precursor to acetylcholine, have also been detected *via* magnetic resonance spectroscopy in thalamic, anterior cingulate, and caudate nucleus regions of antipsychotic-naïve patients [15, 95], possibly indicative of impaired ChAT activity. 

There is also considerable evidence of muscarinic acetylcholine receptor (mAChR) alterations (particularly M_1_ and M_2_) in schizophrenia. These mAChR subtypes are known to play important roles in several signaling pathways that modulate neuronal excitability, synaptic plasticity, and learning and memory [97, 99]. Further, these mAChR subtypes are the ones expressed in highest quantities in the mammalian brain. Older studies reported increased M_2_/M_4_ receptor binding in the orbital frontal cortex and putamen [70, 100] and decreased binding in frontal, parietal, and temporal cortices [6] in schizophrenic brains, while more recent studies indicate decreases in M_1_/M_4_ receptor binding in the striatum, hippocampal formation, prefrontal cortex, and anterior cingulate cortex [25, 22, 47]. Deficits in M_2_/M_4_ receptors as well as cholinergic interneurons in the striatum of schizophrenic brains have also been reported [21]. 

There is also credible evidence to support the argument that both high and low affinity nicotinic acetylcholine receptors (nAChRs) are diminished in schizophrenia. High-affinity (heteromeric α_4_β_2_ subunit complexes) and low-affinity (homomeric α_7_) nAChRs are ligand (i.e., neurotransmitter) gated ion channels that have been implicated in a variety of learning and memory processes [52]. In mammalian brain, the α_4_β_2_ nAChR subtype of high-affinity nAChR predominates in density and distribution compared with other subtypes [75]. Several comparative autoradiographic studies of postmortem tissues from schizophrenic patients indicate decreased α_4_β_2_ nAChR binding in several brain regions including the striatum, hippocampus, and cortex compared with controls [11, 27, and review,  33]. There is also significant evidence of alterations in low affinity α_7 _nAChR deficits in schizophrenia. α_7 _nAChRs comprise approximately 10% of the total number of nAChRs in mammalian brain, but modulate a variety of calcium-dependent events in neurons including neurotransmitter release [37, 59], postsynaptic signaling [17, 41] and neuronal survival [7, 62]. Evidence of α_7 _nAChR alterations in schizophrenia includes postmortem deficits in the hippocampus and frontal cortex [38] and linkage analyses implicating chromosome 15q14 (the region that includes the α_7_ neuronal nAChR gene). Polymorphisms in the core promoter of the α_7_ neuronal nAChR gene (CHRNA7; GeneBank accession no. Z23141) are associated altered P50 evoked responses to repeated auditory stimuli (i.e., indicative of sensory gating abnormalities) in schizophrenia [reviewed,^ 32^]. α_7_ nAChR deficits may also contribute to other abnormalities in schizophrenia including deficits in smooth pursuit eye movements, sustained attention, and cognition [reviewed,^ 57^]. Collectively, these findings have lead to the suggestion that a reduction in α_7_ nAChR expression could potentially serve as a biomarker for schizophrenia [34, 76].

## RELEVANT NEUROTROPHINS

The important role of neurotrophins in the pathophysiology and pharmacotherapy of schizophrenia is gaining interest [14, 85]. Neurotrophins, including nerve growth factor (NGF) and brain-derived neurotrophic factor (BDNF), and their respective receptors serve important roles in neurodevelopment [58], while, in the mature nervous system, they promote neuronal survival and modulate synaptic plasticity of dopaminergic, serotonergic, and interestingly, cholinergic neurons [30, 23, 50]. Like NGF [20, 51], BDNF promotes survival and differentiation of basal forebrain cholinergic neurons [29], stimulates the release of acetylcholine [49] and supports the maintenance of synaptic plasticity and cognitive processes [67]. To date, however, only a relatively few studies have been designed to investigate the potential role of neurotrophin alterations in schizophrenia. Bersani and collaborators [9] reported diurnal plasma fluctuations in NGF (i.e., higher levels in the morning and lower levels in the evening in schizophrenics, compared to controls), while Parikh *et al.*, 2003 [71] detected lower plasma NGF levels (compared to controls) in first episode, never medicated schizophrenic patients, as well as chronically-treated patients. Regarding BDNF, in one postmortem study, significant increases in cortical areas and significant decreases in the hippocampus were detected in schizophrenic patients compared with controls. [28]. Takahashi *et al*., 2000 [87], detected elevated levels of BDNF specifically in the anterior cingulate cortex and hippocampus of schizophrenic patients, while the BDNF receptor TrkB was reduced in the hippocampus and prefrontal cortex. In another study, both BDNF and TrkB mRNA levels were significantly decreased in the prefrontal cortex of subjects with schizophrenia [40].

The effects of antipsychotics on neurotrophins are even less well-understood and, to date, most conclusions rely on animal studies. In rodents, both acute (3 days) and subchronic (21 days) exposure to haloperidol decreased the levels of BDNF in the prefrontal cortex, hippocampus, and amygdala. Decreased BDNF concentrations [4] and decreased expression of BDNF mRNA [54] were also observed after risperidone and clozapine administration. Angelucci and collaborators [4] reported that subchronic (29 day) treatment with haloperidol or risperidone in rats increased NGF immunoreactivity in the hypothalamus, but decreased levels in the striatum and hippocampus. These researchers more recently [5] found that 29 days of oral olanzapine treatment increased NGF in the hippocampus, occipital cortex and hypothalamus, but decreased BDNF in the hippocampus and frontal cortex. The results of recent studies in our laboratory indicate that the effects of conventional and second generation antipsychotics on NGF are temporally dependent, specifically; they can be very different depending on the length of time of administration. Specifically, while NGF levels in the hippocampus were either unchanged or upregulated in rats in response to several antipsychotics during short periods of treatment (7, 14, and 45 days) they were significantly decreased by haloperidol, chlorpromazine, olanzapine, and risperidone when continuously administered orally for 90 or 180 days [78, 90].

## ANTIPSYCHOTICS AND CENTRAL CHOLINERGIC FUNCTION: DELETERIOUS EFFECTS

Taking into consideration the information provided above, an interesting question arises as to what extent antipsychotic drugs affect the central cholinergic system. The conventional antipsychotics, thioridazine and chlorpromazine, and the atypical agents, clozapine and olanzapine bind all of the known muscarinic acetylcholine receptor subtypes with relatively high affinity [reviewed,^ 16^], although the significance of this binding to the therapeutics and adverse effects is unknown. Both conventional and atypical antipsychotics have been observed to increase acetylcholine levels in the hippocampus of rats [84], although the effects of the atypical agents, clozapine and olanzapine, were more robust. Further, the atypical agents, clozapine, olanzapine, risperidone, and ziprasidone significantly increased acetylcholine release in rat medial prefrontal cortex, whereas the haloperidol and thioridazine did not [44]. These differential effects on acetylcholine release were hypothesized (in the studies cited above) to underlie the reported superiority of atypical antipsychotics on cognitive function when compared to conventional agents. However, it is unclear whether such effects would persist for extended periods of time (i.e., the more clinically relevant question regarding antipsychotics). It is also unclear whether such an acute effect would actually be beneficial to cognition; given the fact that muscarinic antagonists such as atropine and scopolamine (i.e., drugs that impair cognition) are well-documented to acutely elevate acetylcholine levels in a number of regions of the brain including the hippocampus and cortex [24, 65].

There is significant evidence to indicate that chronic treatment with antipsychotic drugs alters cholinergic function. It has been known for many years that conventional antipsychotics can produce time-dependent (biphasic) cholinergic responses in the striatum of animals. This effect has been implicated as a potential mechanism of the adverse motor effects of conventional antipsychotic in humans. Specifically, an increase in the activity of cholinergic interneurons in the striatum induced by conventional antipsychotics initially appears to correlate with the extrapyamidal side effects in humans, whereas more prolonged periods of exposure correlate with decreases in cholinergic activity below baseline (i.e., effects that correspond with the emergence of tardive dyskinesia [63]. Such effects may also account for the ability of anticholinergic drugs such as benztropine and diphenhydramine to reduce extrapyamidal side effects of conventional agents during early use, and their lack of efficacy (or tendency to exacerbate symptoms) in tardive dyskinesia. In light of a possible cholinergic basis for antipsychotic-related extrapyramidal effects and TD, another logical question arises as to whether such cholinergic effects might influence information processing and cognition, since cholinergic interneurons in the striatum are now known to exhibit long term potentiation (LTP) and to play an important role procedural learning [48]. We recently detected biphasic; time-dependent effects of both first and second generation antipsychotics in memory-related brain areas such as the hippocampus in rats [90, 91, 94]. In fact, we now have convincing evidence of time-dependent changes resulting from antipsychotics in the vesicular acetylcholine transporter, the cholinergic enzyme, choline acetyltransferase, as well as nicotinic (α_7_) and muscarininc (M_2_) acetylcholine receptors [88, 89, 90, 93]. As in the case of the unexplained mechanisms that underlie the time dependence of the therapeutic effects of antipsychotic drugs (discussed in the Introduction), the mechanisms for the long term antipsychotic-related cholinergic alterations are unknown. We have proposed two potential mechanisms: antipsychotic antagonist activity at dopaminergic-D_2_ receptors on cholinergic neurons (leading to cholinergic dysregulation) and, adverse antipsychotic effects on neurotrophins that support cholinergic neurons such as nerve growth factor and brain derived growth factor (see review [91]). 

## ANTIPSYCHOTICS AND CENTRAL CHOLINERGIC FUNCTION: THERAPEUTIC IMPLICATIONS

Reports of cholinergic deficits in schizophrenia (described above), have given rise to cholinergic strategies for improving both the psychotic symptoms and cognitive deficits in schizophrenia. More than 50 years ago Pfeiffer and Jenney [77] reported antipsychotic effects of the cholinomimetic compound arecoline in schizophrenic patients. Further, acetylcholinesterase inhibitors (e.g., donepezil, galantamine) [reviewed,^ 103^] and the muscarinic M_1_/M_4_ preferring receptor agonist, xanomeline have been reported to improve behavioral symptoms in Alzheimer's disease patients [10]. This information combined with the aforementioned cholinergic deficits observed in schizophrenia has provided the basis for the idea of evaluating compounds such as acetylcholinesterase inhibitors (AChEIs), mAChR agonists, nAChR agonists, or allosteric activators of mAChR and nAChRs as therapeutic agents in schizophrenia [reviewed,^ 33^,^ 19^]. Among these approaches, one that appears to be gaining momentum is the evaluation of mAChR agonists and allosteric activators of mAChRs. Neurochemical and behavioral studies in *mAChR*-mutant mice indicate that M_1_ and/or M_4_ receptors (in particular) might serve as important therapeutic targets for schizophrenia [reviewed,^ 101^]. Accordingly, the M_1_/M_4_ preferring muscarinic agonist, xanomeline (in addition to its positive effects on the behavioral symptoms of AD noted above) demonstrates an antipsychotic-like profile in the rat [86] and it has been shown to improve both positive and negative symptoms in schizophrenic patients (study published in abstract form, [83]). Most recently, highly selective allosteric potentiators of the M_4_ mAChR (e.g., VU10010) and the M_1_ mAChR (TBPB) have been developed and shown to have robust effects in several animal models commonly used to predict antipsychotic activity [reviewed,^ 19^]. Clinically, the approach that has been evaluated most extensively to date (i.e., and published) has been the adjunctive administration of AChEIs (i.e., with antipsychotics) to improve cognitive function. The data from some of the studies have been equivocal; the beneficial cognitive effects of donepezil or rivastigmine as add-on treatments observed in preliminary open-label studies and case reports, were not confirmed in randomized, double-blind, and placebo controlled studies [31, 82]. More recently, a small, randomized, double-blind clinical trial (N=8) indicated that adjunctive treatment with the AChEI, galantamine, improved short-term memory and attention in schizophrenic or schizoaffective patients who were stabilized on risperidone [81]. Most recently, 86 schizophrenia patients were evaluated in a 12-week double-blind, placebo-controlled, randomized clinical trial with 42 subjects assigned to galantamine and 44 patients assigned to placebo [13]. The treatment effect for the overall composite score was not significant; however, follow-up analyses indicated improvements in processing speed and verbal memory. While such data are encouraging, additional studies will be required to verify the validly of adjunctive cholinesterase inhibitors as a reliable therapeutic approach to cognition enhancement.

As we have reviewed previously [91] there may be several factors that underlie the equivocal nature of the studies cited above, such as cigarette smoking (and thus the confounding cholinergic receptor effects of nicotine) by the research subjects, exposure to other drugs of abuse, differences in the neuropsychological measures employed, etc. Interestingly, smoking rates in the schizophrenic population in North America have been reported to approach 90 percent in some studies [43], i.e., more that three times the rate of the general population and considerably higher than for the rate observed in other psychiatric conditions [26, 96]. A variety of hypotheses have been presented to explain this phenomenon including volitional (i.e., self-medication) attempts to overcome certain schizophrenia symptoms such as depression, anxiety, and anhedonia (see [69] for review), nAChR deficit-related impairments of information processing, psychomotor speed, and cognition [1, 53] as well as to reduce antipsychotic-related side effects. There is evidence to support some of these hypotheses. Nicotine (for example) has been shown in a variety of animal and human studies to have anxiolytic effects ﻿ [79, 12], to improve information processing and cognition [53] and to ameliorate some side-effects (e.g., akathisia, bradykinesia) associated with first generation antipsychotics [2, 104].

An important factor that could certainly influence responses to the adjunctive agent is the unique medication history of the study subject. Specifically, factors such as the history of antipsychotic drug exposure, the particular antipsychotic agent currently being administered, and how long the patient has been exposed to a particular antipsychotic, could be especially important to the adjunctive treatment response. In our chronic animal studies [88, 92], we have found that several antipsychotics can lead to decreases in specific cholinergic markers (e.g., α_7_ nAChRs in association with haloperidol and risperidone). Such findings may indicate that a more patient specific (i.e., tailored) therapeutic approach (using specific types of cholinergic agents) that takes into account the unique medication history (and current antipsychotic therapy) of the patient may be necessary to improve the symptoms of schizophrenia

## CONCLUSIONS

The therapeutics of schizophrenia are particularly challenging given the heterogeneity of disease symptoms and our relatively poor understanding of the neuropathology. In order to effectively address the core features of the illness (i.e., positive, negative, and cognitive symptoms), novel strategies need to be designed that take in to account a number of import patient-specific factors. These factors include the specific symptom profile, the unique medication history of the particular patient, and concurrent exposure to other drugs (e.g., nicotine). Reports of alterations in the brain cholinergic system in schizophrenic patients combined with animal data suggesting that chronic exposure to antipsychotics can lead to cholinergic alterations, provides the impetus for investigating both primary and adjunctive cholinergic strategies to the pharmacotherapy of schizophrenia.
